# Gene Expression Profiling in Dermatitis Herpetiformis Skin Lesions

**DOI:** 10.1155/2012/198956

**Published:** 2012-09-06

**Authors:** M. Dolcino, E. Cozzani, S. Riva, A. Parodi, E. Tinazzi, C. Lunardi, A. Puccetti

**Affiliations:** ^1^Instituto Giannina Gaslini, Clinical Immunology Unit, 16148 Genoa, Italy; ^2^Section of Dermatology, Department of Health Sciences, University of Genoa, 16132 Genoa, Italy; ^3^Section of Internal Medicine, Department of Medicine, University of Verona, 37134 Verona, Italy; ^4^Section of Histology, Department of Experimental Medicine, University of Genoa, Via Marsano 10, 16132 Genoa, Italy

## Abstract

Dermatitis herpetiformis (DH) is an autoimmune blistering skin disease associated with gluten-sensitive enteropathy (CD). In order to investigate the pathogenesis of skin lesions at molecular level, we analysed the gene expression profiles in skin biopsies from 6 CD patients with DH and 6 healthy controls using Affymetrix HG-U133A 2.0 arrays. 486 genes were differentially expressed in DH skin compared to normal skin: 225 were upregulated and 261 were downregulated. Consistently with the autoimmune origin of DH, functional classification of the differentially expressed genes (DEGs) indicates a B- and T-cell immune response (LAG3, TRAF5, DPP4, and NT5E). In addition, gene modulation provides evidence for a local inflammatory response (IL8, PTGFR, FSTL1, IFI16, BDKRD2, and NAMPT) with concomitant leukocyte recruitment (CCL5, ENPP2), endothelial cell activation, and neutrophil extravasation (SELL, SELE). DEGs also indicate overproduction of matrix proteases (MMP9, ADAM9, and ADAM19) and proteolytic enzymes (CTSG, ELA2, CPA3, TPSB2, and CMA1) that may contribute to epidermal splitting and blister formation. Finally, we observed modulation of genes involved in cell growth inhibition (CGREF1, PA2G4, and PPP2R1B), increased apoptosis (FAS, TNFSF10, and BASP1), and reduced adhesion at the dermal epidermal junction (PLEC1, ITGB4, and LAMA5). In conclusion, our results identify genes that are involved in the pathogenesis of DH skin lesions.

## 1. Introduction 

Dermatitis herpetiformis (DH) is an autoimmune subepidermal blistering skin disease characterized by intense pruritic papulovesicular eruptions mainly localized on extensor surfaces [1]. DH typically develops in patients with celiac disease (CD). The two conditions share the same genetic background (HLA genes DQ2–DQ8), improve following a gluten-free diet (GFD), and are mediated by IgA autoantibodies [2]. IgA antibodies against tissue transglutaminase (tTG) are detectable both in CD and DH, while autoantibodies directed against epidermal transglutaminase (eTG) are a typical serological marker of patients with DH [3].

The key feature of DH is a granular deposition of IgA within the tips of dermal papillae and along the basement membrane of perilesional skin. eTG has been shown to colocalize with such IgA deposits [4]. Typical histopathologic features of DH consist of accumulation of neutrophils and a few eosinophils with formation of papillary microabscesses which then coalescence to form a subepidermal bulla. 

Moreover, a perivascular cellular infiltrate composed mainly by CD4+ lymphocytes is also present [5].

In DH, blister formation is associated with epidermal splitting due to destruction of basement membrane components and proteolysis of adhesion molecules at the dermal epidermal junction. A comprehensive analysis of the molecular mechanisms that coordinate the initiation and progression of the pathological process is still lacking. Our approach consists in the use of a gene array strategy that allows the simultaneous detection of thousands of genes in a given sample. We have examined gene expression directly in the skin tissue of patients with DH to analyze the transcriptional events that culminate in the skin lesion formation. We report here patterns of transcripts in 6 DH patients using DNA microarrays that characterize injured skin and identify signatures of gene expression that are involved in the pathogenesis of blister formation. The analysis of modulated genes provides evidence for the intervention of genes involved in immune activation, inflammation, impaired adhesion and cell death, considered key features in the pathogenesis of the disease.

## 2. Materials and Methods

### 2.1. Patients

Six adult patients (3 men and 3 females; mean age 51 years, median age 52 years, and age range 36–59 years) with DH and CD, showing all clinical and immunopathological features of the diseases, were included in this study. All patients had the typical clinical features of DH, with erythematous papules and vesicles symmetrically distributed on the extensor surfaces of the upper and/or lower extremities and buttocks. The duodenal histological damage of the 6 patients at diagnosis ranged from grade 2 to 3b, according to Marsh's classification [6]. In particular, three patients had a grade 2 damage, and three patients had grade 3b damage. Five out of six patients suffered from gastrointestinal symptoms (diarrhoea, abdominal distension, and pain); one patient had extraintestinal symptoms (iron deficiency anaemia and weight loss).

Skin biopsies presented classical histopathologic features of DH, including subepidermal cleft with neutrophils and/or eosinophils at the tips of the dermal papillae and granular deposits of IgA at the tips of derma papillae on direct immunofluorescence.

Serologically, five out of six patients had serum anti-tTG and antiendomysium (EMA) IgA antibodies without gluten-free diet. The seronegative patient had a duodenal biopsy with a grade 3b histological damage and was affected by IgA deficiency. Indeed anti-tTG IgG were detected in this patient.

All patients were on normal gluten-containing diet and were not taking Dapsone at the moment of skin biopsy. Two punch biopsies of 6 mm each were performed at the diagnosis on each one of the 6 patients from early lesional skin (grouped erythematous papules surmounted by vesicles) following local anaesthesia (1% lidocaine with 1/100,000 epinephrine). Skin specimens for biopsy were obtained from elbows (2 patients) and from buttocks (4 patients).

Normal skin biopsies were obtained from 6 sex- and age-matched healthy adult subjects (3 males and 3 females, mean age 50 years, median age 53 years, age range 34–60 years) with no evidence of gastrointestinal or skin disease. Specimens were snap-frozen in liquid nitrogen immediately after biopsy. 

All the subjects (patients and controls) were of Caucasian origin from Northwestern Italy.

The patients included showed no evidence of other coexisting autoimmune diseases.

Written informed consent was obtained in each case. The study was conducted according to the Declaration of Helsinki Principles and was approved by the local ethical committee.

### 2.2. Samples Preparation

Tissue samples from every single patient were frozen in liquid nitrogen immediately after dissection and stored at −70°C until homogenization. Frozen samples were homogenized in TRI REAGENT (1 mL per 50–100 mg of tissue) in a Potter-type mechanical homogenizer with Teflon pestle. RNA extraction, preparation of cRNA hybridization, and scanning of probe arrays for each samples were performed according to the protocols of the manufacturer (Affymetrix, Santa Clara, CA, United States) by Cogentech Affymetrix microarray unit (Campus IFOM-IEO, Milan, Italy) using the human genome U133A 2.0 gene chip (Affymetrix). The human genome U133A gene chip is a single array representing 14,500 well-characterized human genes and including more than 22,000 probe sets and 500,000 distinct oligonucleotide features.

### 2.3. Gene-Array Analysis

The different gene expression patterns were analyzed by using Gene Spring software, version 11.0 (Agilent Technologies, Santa Clara, CA, United States).

The normalized background-corrected data were transformed to the log_2_ scale. A signal log_2_ ratio of 1.0 indicates an increase of the transcript level by twofold change (2 F.C.), and −1.0 indicates a decrease by twofold (−2 F.C.). A signal log_2_ ratio of zero would indicate no change.

The unpaired  *t*-test was performed to determine which genes were modulated at a significance level (*P* < 0.05), and *P* values were corrected for multiple testing by using Bonferroni correction.

Finally, statistically significant genes were selected for final consideration when their expression was at least 1.5-fold different in the test sample versus control sample.

 Genes that passed both the *P* value and the F.C. restriction were submitted to a functional classification according to the Gene Ontology (GO) annotations (http://www.geneontology.org/).

## 3. Results

In order to identify genes involved in the pathogenesis of the typical skin lesions of DH, the gene expression patterns of 6 skin biopsies from 6 patients affected by DH were compared with 6 skin biopsies from 6 healthy controls.

A  *P*  value criterion (*P* < 0.05) and a fold change criterion (FC > 1.5) were both applied to the signal variation of every single gene to select robust and statistically significant changes between baseline and experimental arrays.

For statistical comparison, an unpaired  *t*-test was calculated, and after a Bonferroni correction, 1191 transcripts resulted statistically significantly modulated (*P* < 0.05).

Among these transcripts, 486 also fulfilled the fold change criterion, since they were differentially expressed 1.5 fold or more; in particular 225 and 261 transcripts resulted, respectively, to be up- and downregulated.

Such transcripts were classified in functional categories according to Gene Ontology annotations, including immune response, apoptosis, cell growth, proliferation and differentiation, inflammatory response, production and remodelling of the extracellular matrix, and metabolism.

Table 1 shows a detailed representation of genes within the above-mentioned clusters. The table also includes GeneBank accession numbers and fold changes.

Among genes involved in the immune response, upregulated genes play a role in T lymphocyte activation, for example, lymphocyte-activation gene 3 (LAG3) [7] and dipeptidyl-peptidase 4 (DPP4) [8], or in B and T lymphocyte migration, for example, 5′-nucleotidase and ecto-CD73 (NT5E) [9].

Other upregulated genes involved in the immune response belong to the CD40 signalling pathways, including the TNF receptor-associated factor 5 (TRAF5) or play a role in innate immunity such as the killer cell lectin-like receptor subfamily K, member 1 (KLRK1, better known as NKG2D), or SAM domain and HD domain 1 (SAMHD1) [10].

Moreover, a cluster of genes that have a role in the inflammatory process was upregulated. This cluster encompasses the interferon, gamma-inducible protein 16 (IFI16), bradykinin receptor B2 (BDKRB2), chemokine (C-C motif) ligand 5 (CCL5), ectonucleotide pyrophosphatase/phosphodiesterase 2 (ENPP2, also called autotaxin), interleukin 8 (IL8), prostaglandin F receptor (PTGFR), follistatin-like 1 (FSTL1), selectin L (SELL), selectin E (SELE), thrombospondin 1 (THBS1), and serglycin (SRGN).

Moreover, a downregulation of the diacylglycerol kinase, alpha 80 kDa (DGKA) [11], a negative regulator of the respiratory burst in normal polymorphonuclear cells, and of calpain 3 (CAPN3) that downregulates cell migration in resting monocytes, was observed.

Many genes coding for protein involved in apoptosis and/or in apoptosis regulation resulted to be modulated in pathological samples. Among these, several proapoptotic genes were upregulated such as TNF receptor superfamily, member 6 (FAS), tumour necrosis factor (ligand) superfamily, member 10 (TNFSF10) brain abundant, membrane-attached signal protein 1 (BASP1), stress 70 protein chaperone microsome associated (STCH) [12], serine/threonine kinase 17a (STK17A), G1 to S phase transition 1 (GSPT1) and interferon, and alpha-inducible protein 27 (IFI27) [13].

On the other hand, genes coding for the antiapoptotic protein arginine methyltransferase 5 (PRMT5) and DNA-damage-inducible transcript 4 (DDIT4) were downregulated.

Antiproliferative genes were upregulated in DH skin samples including the cell growth regulator with EF-hand domain 1 (CGREF1) and the tumor suppressor genes named proliferation-associated 2G4 (PA2G4/EBP1) [14].

Moreover positive regulators of cell growth, such as MMS19 nucleotide excision repair homolog (MMS19) and v-erb-b2 erythroblastic leukemia viral oncogene homolog 3 (ERBB3), resulted downregulated.

Several genes involved in extracellular matrix components synthesis as well as in wound healing and tissue repair were upregulated. 

These genes are involved in the synthesis of collagen such as procollagen-lysine, 2-oxoglutarate 5-dioxygenase 2 (PLOD2), or in the production of hyaluronan as hyaluronan synthase 1 (HAS1) [15].

Four genes coding for different collagen molecules were also upregulated, and these are collagen, type I, alpha 2 (COL1A2), collagen, type III, alpha 1 (COL3A1), collagen, type IV, alpha 1 (COL4A1), and collagen, type V, alpha 2 (COL5A2).

Moreover, we also observed an up-regulation of versican (VCAN) [16] and fibrillin 1 (FBN1) genes.

When we analyzed genes involved in extracellular matrix remodeling, we observed an upregulation of several proteases such as matrix metallopeptidase 3 (MMP3) [17], matrix metallopeptidase 9 (MMP9), ADAM metallopeptidase domain 9 (ADAM9), ADAM metallopeptidase domain 19 (ADAM19), plasminogen activator, urokinase (PLAU) [18], and its receptor PLAUR.

Moreover, among proteolytic enzymes, we found an increased expression of genes coding for proteins that belong to the neutrophil and mast cell secretory repertoire such as tryptase alpha/beta 1 (TPSAB1), tryptase beta 2 (TPSB2), chymase 1 (CMA1), carboxypeptidase A3 (CPA3), elastase 2 (ELA2), and cathepsin G (CTSG).

On the contrary, the alpha-1 antiproteinase (SERPINA3) and the metallopeptidase inhibitors 3 and 4 (TIMP3 and TIMP4) were downregulated.

Three genes coding for protein that are present at the dermal-epidermal junctions were downregulated. These transcripts are plectin 1, intermediate filament binding protein 500 kDa (PLEC1) [19], integrin, beta 4 (ITGB4), and laminin, alpha 5 (LAMA5).

## 4. Discussion

Despite the huge effort in elucidating the pathogenesis of DH, a detailed understanding of the molecular events involved in DH lesion formation is still lacking. In the present work we provide for the first time a comprehensive analysis of the transcriptome within DH lesional skin.

First of all, we observed the modulation of genes, that are involved in the regulation of both immune response and inflammation.

Consistently with the autoimmune origin of DH, we found an overexpression of genes involved in T and B immune response (LAG3, TRAF5, DPP4, and NT5E) [7–9]. 

Lymphocyte activation gene-3 (LAG-3; CD223) is a negative costimulatory receptor that modulates T-cell homeostasis, proliferation, and activation; it is a CD4 homolog that is required for maximal regulatory T-cell function and for the control of CD4(+) and CD8(+) T cell. Interestingly, it may be required for the control of autoimmunity [20, 21]. In this setting, the overexpression of LAG-3 can be considered a mechanism to control the autoimmune response.

Many proinflammatory genes were found to be upregulated in DH samples and some of them with high fold changes (Table 1). These transcripts included IFI16, a gene that is activated by oxidative stress and mediates ICAM-1 stimulation by TNF-alpha [22], FSTL1, a proinflammatory protein enhancing IFN-gamma pathway [23, 24], PTGFR, the receptor of prostaglandin F2 alpha that is thought to be increased in skin blisters of DH [25], and the chemokine CCL5 and the bradykinin receptor BDKRB2, both involved in inflammatory cell recruitment and proinflammatory cytokine production [26].

Particular attention deserves the upregulation of selectin-E (SELE) and IL8 (Table 1); indeed Hall et al. [27] demonstrated that patients with DH have an increased serum level of IL-8 that is associated with cutaneous endothelial cell activation and increased expression of SELE [28]. IL-8 triggers inflammatory leukocyte recruitment as well as angiogenesis and cell proliferation [29, 30]. Human neutrophils are the major components of DH inflammatory infiltrate and are able to produce high levels of IL-8 in response to various inflammatory stimuli. Their ability to firmly adhere to the endothelium prior to roll and extravasate into tissue requires the expression of adhesion proteins such as SELE that are expressed at low level on resting endothelial cell surfaces [31, 32]. Interestingly, SELE is upregulated in DH skin samples indicating local endothelial cell activation.

Hall et al. [28] hypothesized that the presence of mucosal inflammation in the gut of patients with DH may be critical in priming both neutrophils and cutaneous endothelial cells through the production of elevated levels of proinflammatory cytokines such as IL8. Our results indicate also a local production of IL8 most probably released by activated neutrophils.

Interestingly, we found overexpression of ENPP2/autotaxin, a molecule that exacerbates inflammation by increasing chemotaxis through the upregulation of neutrophil integrins [33]. We also observed an increased expression of SRGN/serglycin that is important for the retention of key inflammatory mediators inside neutrophil storage granules and secretory vesicles [34].

The downregulation of the two anti-inflammatory genes, DGKA and CAPN3, may be also linked to increased neutrophil migration [35].

Apoptosis is thought to play a role in the pathogenesis of cutaneous lesions, and increased apoptotic events in basal and suprabasal keratinocytes were observed within lesional and perilesional skin of DH [36]. Consistently with this observation, we found overexpression of proapoptotic genes such as FAS and TNFSF10/TRAIL and downregulation of two antiapoptotic transcripts, namely, PRMT5 and DDIT4. These genes may be correlated also to the unique form of apoptotic cell death of neutrophils, called “NETosis,” that has been recently associated with autoimmune phenomena in systemic lupus erythematosus and possibly in other autoimmune diseases [37]. Since neutrophils play a pivotal role in DH skin lesions, we can speculate that NETosis may play a role also in DH.

We noticed a remarkable modulation of genes coding for several components of the extracellular matrix such as collagen type III, IV, and V. An elevated level of collagen type III, IV, and V has been described in the DH blisters of the papillary derma [38]. The gene coding for fibrillin (FBN1) was also upregulated in our DH skin samples. This protein constitutes the major backbone of multifunctional microfibrils in elastic and nonelastic extracellular matrices and may be one of the structural components bound by IgA-reactive deposits in the skin of patients with DH [39].

Matrix degradation at the dermal-epidermal junction has been thought to contribute to DH blister formation [40]. During this assault to the extracellular components, proteases secreted by keratinocytes, macrophages, and neutrophils act in concert. 

We found an increased expression of neutrophil and mast cell enzymes such as TPSB2, TPSAB1, CMA1, CPA3, ELA2, and CTSG that are thought to be involved in the splitting up of epidermis from dermis [41]. Noteworthy high levels of ELA2 have been described in vesicle fluid obtained from patients with DH [42].

Proteases secreted by granulocytes and mast cells could mediate the development of DH cutaneous lesions either directly or indirectly by the activation of metalloproteases [43].

Several genes coding for metalloproteases resulted upregulated in our DH skin samples including MMP3/stromelysin, MMP9/gelatinase B, ADAM9/meltrin gamma, and ADAM19/meltrin beta. It has been demonstrated that MMP3 participates to blister formation by degrading basement membrane components [17].

Airola et al. reported an increased secretion of this enzyme by basal keratinocytes surrounding neutrophil abscesses [17]. MMP9 is another molecule produced by eosinophils and neutrophils that are attracted to the basement membrane zone by integrins and selectins. 

It has been suggested that the formation of blisters may be induced by an overexpression of local enzymes [43], and indeed the results of our gene array experiments indicate an increased production of proteolytic enzymes within the skin lesions. Macrophage metalloelastase (MMP12) was found abundantly expressed in subepithelial macrophages of DH skin lesions by *in situ* hybridisation [44]. The MMP12 transcript was also detected in all our samples; however, only in 4/6 DH samples, the level of expression of this enzyme was significantly upregulated when compared to the controls. For this reason, MMP12 has not been included in the list of upregulated genes. This discrepancy could be ascribed to the different detection methods used (*in situ* hybridisation versus gene array). In the paper by Salmela et al. [44] the increased mRNA expression of MMP12 was confined to subepithelial macrophages. This increase may be diluted in mRNA samples derived from the total biopsy specimen composed by a large number of different cell types. Moreover, the relative content of macrophages may vary in the different skin biopsies used for the gene array analysis.

Interestingly we found a strong downregulation of genes coding for tissue inhibitors of proteases such as SERPINA3, TIMP3 and TIMP4.

Therefore, our gene analysis confirms that an important role in the maintenance and amplification of the immunological processes underlying blister formation may be played by an imbalance between the activities of MMPs and their tissue inhibitors, as previously hypothesised by Zebrowska et al. [45].

Another molecule involved in the degradation of basement membrane is the plasminogen activator urokinase (PLAU) that has been found to be highly expressed in keratinocytes in experimentally induced DH lesions [18]. This molecule may also have an activating role in MMP9 in early phase of blister formation [43]. Interestingly, PLAU and its receptor PLAUR were upregulated in our DH skin samples; moreover, we found an increased expression of thrombospondin 1 (THBS1). THBS1 can downregulate PAI, the inhibitor of plasminogen activator [46], thus eventually reinforcing the final physiological effect of the PLAU overexpression.

It is tempting to speculate that an overexpression of PLAU may lead to increased production of plasmin that in turn activates MMP9, as seen in experiments carried out in mice [47].

We also observed a downregulation of genes coding for proteins involved in the network that anchor the keratin filaments of cells cytoskeleton to the underlying dermis at the dermal-epidermal junctions. These molecules are: PLEC/plectin 1, ITGB4, and LAMA5/laminin alpha 5.

Plectin is a large 200 nm long protein found in hemidesmosomes and whose function is to bind keratin intermediate filaments to the hemidesmosome, and specifically to transmembrane collagen XVII and *β*4 integrins [19]. It has been demonstrated that a defective expression of plectin/HD1 may predispose to blister formation in human skin [48].

Laminin 5 is essential for adhesion of keratinocytes to basement membrane [18], and integrins such as ITGB4 are the main laminin receptor [49].

In DH skin lesions, proteins within the dermal epidermal junction are target of proteolytic enzymes released by neutrophils. In addition, the decreased expression of the above-mentioned molecules might worsen the damage induced by granulocytic enzymatic activity.

Overall, the results obtained support the hypothesis that during blister development, the inflammatory reaction evoked by the autoimmune response typical of the disease is associated to a local overexpression of proteolytic enzymes leading to the detachment of the dermal-epidermal junction. The consequent tissue damage may be amplified by a reduced production of protease inhibitors. 

Moreover, our data suggest that an increased rate of apoptosis and a reduced expression of anchoring proteins at dermal-epidermal junction are key features in DH skin lesions. 

In conclusion, we believe that our study on gene expression gives a better understanding of the molecular mechanisms involved in the pathogenesis of skin lesions in DH. 

## Figures and Tables

**Table 1:** Annotated genes differentially expressed in DH versus healthy controls grouped according to their function.

Functional class	Probe set ID	F.C.	Regulation	Gene symbol	Gene title	Accession number
Immune response	206486_at	1.5	Up	LAG3	Lymphocyte-activation gene 3	NM_002286
	204352_at	1.6	Up	TRAF5	TNF receptor-associated factor 5	NM_004619
	205821_at	1.7	Up	KLRK1	Killer cell lectin-like receptor subfamily K, member 1	NM_007360
	203717_at	2.4	Up	DPP4	Dipeptidyl peptidase 4	NM_001935
	203939_at	3.8	Up	NT5E	5′-nucleotidase, ecto (CD73)	NM_002526
	204502_at	2.0	Up	SAMHD1	SAM domain and HD domain 1	NM_015474

Inflammation	206332_s_at	3.1	Up	IFI16	Interferon, gamma-inducible protein 16	NM_005531
	217738_at	2.0	Up	NAMPT	Nicotinamide phosphoribosyltransferase	NM_005746
	203176_s_at	2.2	Up	TFAM	Transcription factor A, mitochondrial	NM_003201
	205870_at	2.2	Up	BDKRB2	Bradykinin receptor B2	NM_000623
	204655_at	2.2	Up	CCL5	Chemokine (C-C motif) ligand 5	NM_002985
	209392_at	2.3	Up	ENPP2	Ectonucleotidepyrophosphatase	L35594
	202859_x_at	2.3	Up	IL8	Interleukin 8	NM_000584
	211272_s_at	2.4	Down	DGKA	Diacylglycerol kinase, alpha 80 kDa	AF064771
	207177_at	2.5	Up	PTGFR	Prostaglandin F receptor	NM_000959
	208782_at	2.9	Up	FSTL1	Follistatin-like 1	BC000055
	204563_at	7.3	Up	SELL	Selectin L	NM_000655
	206211_at	4.3	Up	SELE	Selectin E	NM_000450
	217800_s_at	1.9	Up	NDFIP1	Nedd4 family interacting protein 1	NM_030571
	214475_x_at	2.8	Down	CAPN3	Calpain 3, (p94)	AF127764
	201859_at	3.1	Up	SRGN	Serglycin	NM_002727
	201110_s_at	5.2	Up	THBS1	Thrombospondin 1	NM_003246

Apoptosis	202558_s_at	1.5	Up	STCH	Stress 70 protein chaperone	NM_006948
	217786_at	1.5	Down	PRMT5	Protein arginine methyltransferase 5	NM_006109
	204781_s_at	1.5	Up	FAS	TNF receptor superfamily, member 6	NM_000043
	202693_s_at	1.7	Up	STK17A	Serine/threonine kinase 17a	NM_004760
	201912_s_at	2.6	Up	GSPT1	G1 to S phase transition 1	NM_002094
	202887_s_at	2.6	Down	DDIT4	DNA-damage-inducible transcript 4	NM_019058
	202687_s_at	2.9	Up	TNFSF10	Tumor necrosis factor (ligand) superfamily, member 10	NM_003810
	202411_at	3.1	Up	IFI27	Interferon, alpha-inducible protein 27	NM_005532
	202391_at	3.1	Up	BASP1	Brain abundant, membrane-attached signal protein 1	NM_006317

Cell proliferation	208676_s_at	1.5	Up	PA2G4	Proliferation-associated 2G4, 38 kDa	U87954
	205937_at	1.5	Up	CGREF1	Cell growth regulator with EF-hand domain 1	NM_006569
	1773_at	1.5	Down	FNTB	Farnesyltransferase, CAAX box, beta	L00635
	202886_s_at	2.2	Up	PPP2R1B	Protein phosphatase 2, regulatory subunit A, beta isoform	M65254
	202167_s_at	1.9	Down	MMS19	MMS19 nucleotide excision repair homolog	NM_022362
	203108_at	2.1	Up	GPRC5A	G protein-coupled receptor, family C, group 5, member A	NM_003979
	202454_s_at	2.7	Down	ERBB3	v-erb-b2 erythroblastic leukemia viral oncogene homolog 3	NM_001982

	204798_at	1.6	Up	MYB	v-myb myeloblastosis viral oncogene homolog	NM_005375
	218717_s_at	1.7	Up	LEPREL1	Leprecan-like 1	NM_018192
	209765_at	1.8	Up	ADAM19	ADAM metallopeptidase domain 19	AF311317
	202381_at	1.8	Up	ADAM9	ADAM metallopeptidase domain 9	NM_003816
	203044_at	2.1	Up	CHSY1	Chondroitin sulfate synthase 1	NM_014918
Extracellular matrix	205479_s_at	2.1	Up	PLAU	Plasminogen activator, urokinase	NM_002658
	210845_s_at	2.1	Up	PLAUR	Plasminogen activator, urokinase receptor	U08839
	201995_at	2.2	Up	EXT1	Exostoses (multiple) 1	NM_000127
	205828_at	3.4	Up	MMP3	Matrix metallopeptidase 3 (stromelysin 1)	NM_002422
	203936_s_at	2.2	Up	MMP9	Matrix metallopeptidase 9	NM_004994
	202620_s_at	2.4	Up	PLOD2	Procollagen-lysine, 2-oxoglutarate 5-dioxygenase 2	NM_000935
	207316_at	2.8	Up	HAS1	hyaluronan synthase 1	NM_001523
	203343_at	3.2	Up	UGDH	UDP-glucose dehydrogenase	NM_003359
	204620_s_at	4.0	Up	VCAN	Versican	NM_004385
	202766_s_at	5.6	Up	FBN1	Fibrillin 1	NM_000138
	202404_s_at	4.1	Up	COL1A2	Collagen, type I, alpha 2	NM_000089
	201852_x_at	2.9	Up	COL3A1	Collagen, type III, alpha 1	NM_000090
	211980_at	2.4	Up	COL4A1	Collagen, type IV, alpha 1	NM_001845
	221730_at	2.7	Up	COL5A2	Collagen, type V, alpha 2	NM_000393
	207134_x_at	2.2	Up	TPSB2	Tryptase beta 2	NM_024164
	210084_x_at	2.1	Up	TPSAB1	Tryptase alpha/beta 1	AF206665
	214533_at	3.5	Up	CMA1	Chymase 1, mast cell	NM_001836
	205624_at	2.1	Up	CPA3	Carboxypeptidase A3 (mast cell)	NM_001870
	206871_at	3.3	Up	ELA2	Elastase 2, neutrophil	NM_001972
	205653_at	5.0	Up	CTSG	Cathepsin G	NM_001911
	202376_at	1.7	Down	SERPINA3	Serpin peptidase inhibitor, clade A, member 3	NM_001085
	201147_s_at	1.8	Down	TIMP3	TIMP metallopeptidase inhibitor 3	NM_000362
	206243_at	2.8	Down	TIMP4	TIMP metallopeptidase inhibitor 4	NM_003256

Dermal-epidermal junction	216971_s_at	1.5	Down	PLEC1	Plectin 1, intermediate filament binding protein	Z54367
	214292_at	1.5	Down	ITGB4	Integrin, beta 4	AA808063
	210150_s_at	1.5	Down	LAMA5	Laminin, alpha 5	BC003355

Metabolism	207786_at	1.9	Down	CYP2R1	Cytochrome P450, family 2, subfamily R, polypeptide 1	NM_024514
	211019_s_at	2.1	Down	LSS	2,3-oxidosqualene-lanosterol cyclase	D63807
	205676_at	2.5	Up	CYP27B1	Cytochrome P450, family 27, subfamily B, polypeptide 1	NM_000785
